# Reliability and quality of reflux esophagitis educational videos on TikTok and Bilibili: A cross-sectional study

**DOI:** 10.1097/MD.0000000000047565

**Published:** 2026-02-06

**Authors:** Zhenzhen Deng, Yueliang Xie, Shengfeng Wang, Lu Huang

**Affiliations:** a Department of Pharmacy, the Third Xiangya Hospital of Central South University, Changsha, Hunan, China.

**Keywords:** Bilibili, health communication, reflux esophagitis, TikTok, video quality

## Abstract

Social media platforms have become an increasingly important sources for obtaining health information. Short-form videos related to reflux esophagitis (RE) are widely disseminated on Chinese video platforms. The quality and reliability of such content remain unclear. This cross-sectional study analyzed the top 150 RE-related videos from TikTok and Bilibili. Video characteristics, uploader background, and engagement metrics were extracted. Information quality was evaluated using the Global Quality Score (GQS), modified DISCERN and Journal of the American Medical Association benchmark criteria assessment tools. The Mann–Whitney *U* test or Kruskal–Wallis test was applied for subgroup comparisons, and Spearman rank correlation coefficient was used for correlation analysis. A total of 214 videos met the inclusion criteria, and the overall quality was moderate. The median GQS score was 3 (IQR 2–3), the median modified DISCERN score was 2 (IQR 2–3), and the median Journal of the American Medical Association score was 1 (IQR 1–2). TikTok videos had significantly higher GQS scores than those on Bilibili (*P* < .05). Videos uploaded by gastroenterologists received the highest GQS scores (*P* < .05). Clinical manifestations were the most frequently discussed topic (75.70%), whereas epidemiology was least represented (13.55%). No significant correlations were found between engagement metrics and quality scores (*P* > .05). This study provides a comprehensive evaluation of RE-related short videos across 2 major social media platforms. Uploader professional background, particularly gastroenterology specialization, was a stronger determinant of information quality than engagement metrics. The findings highlight the limitations of popularity-based indicators for identifying credible medical information and underscore the need to promote specialist participation and evidence-informed governance to improve the quality of digital health communication.

## 1. Introduction

Reflux esophagitis (RE) is an inflammatory condition of the esophagus caused by gastric acid reflux and is clinically characterized by symptoms such as heartburn and regurgitation.^[1]^ As a major manifestation of gastroesophageal reflux disease, RE affects approximately 18% to 25% of patients, and its prevalence has increased steadily worldwide.^[2–4]^ Inadequate or delayed management may result in serious complications, including esophageal stricture, bleeding, Barrett esophagus, and esophageal adenocarcinoma.^[5]^ Although pharmacological therapy remains the mainstay of treatment, surgical intervention is required in refractory cases.^[1,6–8]^ Beyond its clinical burden, RE significantly impairs patients’ quality of life and generates substantial healthcare costs related to long-term management.^[9,10]^

Improving public awareness of RE and enhancing the ability to access reliable health information are therefore essential for early diagnosis, standardized treatment and long-term disease management.^[11]^ In recent years, the rapid digitalization of healthcare communication has transformed how medical information is disseminated, with social media platforms becoming major sources of health-related knowledge for the general public.^[12–15]^ Platforms such as YouTube, TikTok, and Bilibili extend the reach of health education beyond traditional clinical settings by providing intuitive, visual, and easily accessible content.^[16,17]^ Their interactive features further allow rapid dissemination and active audience engagement.

However, the open nature of video platforms introduces substantial variability in content quality.^[18]^ Differences in creators’ professional backgrounds, combined with recommendation algorithms that prioritize engagement over accuracy, may amplify the visibility of incomplete or misleading health information.^[19–21]^ Previous analyses of medical videos on TikTok and Bilibili have consistently reported suboptimal information quality across various conditions, including premature ovarian failure,^[22]^ thyroid eye disease,^[23]^ prostate cancer,^[24]^ knee osteoarthritis,^[25]^ uterine fibroid,^[26]^ radiotherapy,^[27]^ and so on. These findings raise concerns regarding the reliability of healthcare education videos.

Given the clinical importance of RE and the potential consequences of misinformation, a systematic evaluation of RE-related video content is warranted. To date, evidence regarding the quality, reliability and engagement characteristics of RE-related videos on major Chinese video platforms remains limited. Therefore, this study aims to assess the content characteristics and information quality of RE-related videos on TikTok and Bilibili using validated evaluation tools and to examine the relationship between information quality and user engagement. We aim to provide insights that may inform healthcare professionals, patients, and policymakers with promoting the scientific dissemination and effective regulation of health information on video platforms.

## 2. Materials and methods

### 2.1. Search strategy

This study collected video data on the topic of “Reflux Esophagitis” from TikTok (Douyin; https://www.douyin.com/) and Bilibili (https://www.bilibili.com/). On August 28, 2025, the keyword “反流性食管炎” was used to search both platforms. All searches were conducted through a newly registered account. Referring to previous studies,^[24,25,27]^ and to balance content representativeness with evaluation feasibility of the evaluation process, we selected the top 150 videos from each platforms based on their default ranking order.

### 2.2. Inclusion and exclusion criteria

**Figure 1.**
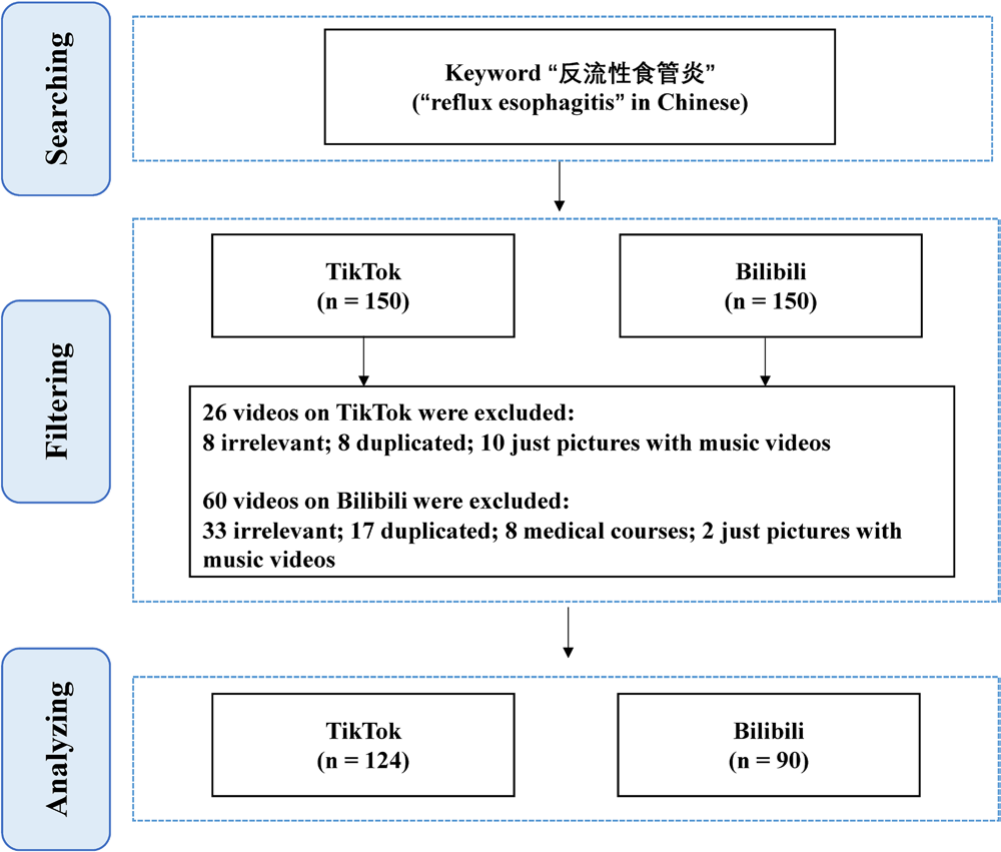
The flowchart of the study for depicting the video selection process.

Inclusion criteria: Chinese language videos and videos incorporating content on RE. Exclusion criteria were: not related to RE; duplicate videos, including exact or near-identical reposts or reuploads of previously analyzed content; academic teaching videos intended for medical students; and image-only videos with background music. The detailed collection procedure is shown in Figure 1.

The following general information was extracted and recorded in Microsoft Excel: video URL, platform (TikTok or Bilibili), video length (seconds), number of likes, comments, collections, and shares. Uploaders were categorized as professionals, nonprofessionals (mainly patients), or institutions. Professionals were further classified as gastroenterologists, traditional Chinese medicine (TCM) physicians, and other specialists.

### 2.3. Video quality and reliability assessment

Three widely recognized tools were used to evaluate the quality and reliability of videos: the Global Quality Score (GQS),^[28]^ the modified DISCERN (mDISCERN) instrument^[29]^ and the Journal of the American Medical Association (JAMA) benchmark criteria.^[30]^ GQS: Videos were scored on a 5-point scale ranging from 1 (poor) to 5 (excellent). mDISCERN consists of 5 criteria, each rated as “yes” (1 point) or “no” (0 point). Videos meeting the criteria were then further evaluated using a 5-item questionnaire, with each item scored from 1 (very poor) to 5 (excellent). The JAMA benchmark criteria assessed authorship, attribution, currency, and disclosure. Detailed descriptions of these instruments are provided in Table S1 to S3, Supplemental Digital Content, https://links.lww.com/MD/R319.

### 2.4. Evaluator training and calibration

Before the formal assessment, 2 primary reviewers with medical backgrounds underwent standardized training on the evaluation instruments, including the GQS, mDISCERN, and JAMA benchmark criteria. Detailed instructions and operational definitions for each scoring item and content domain were reviewed to ensure consistent interpretation. A calibration process was conducted using sample videos that were not included in the final analysis. These videos were independently scored by both reviewers, after which discrepancies were discussed in consensus meetings. During calibration, ambiguous scoring criteria and content coding rules were clarified. After satisfactory agreement was achieved, the reviewers independently evaluated all eligible videos. In cases of disagreement, a senior third reviewer adjudicated the final scores through discussion to reach consensus.

### 2.5. Video content evaluation

Video content was evaluated across 6 domains: epidemiology, etiology, clinical manifestations, diagnosis, treatment, and prognosis. For each domain, scoring was based on the level of information provided: not mentioned (0 point), partially mentioned (1 point), or fully mentioned (2 points). “Partially mentioned” indicated general or fragmented information lacking sufficient clinical context, whereas “fully mentioned” required minimal disease-specific completeness, providing coherent and non-misleading explanations suitable for a general audience. Detailed definitions and examples for each domain were specified in the coding manual (Table S4, Supplemental Digital Content, https://links.lww.com/MD/R319). For treatment content, we recorded whether drug therapy and surgical treatment were mentioned. Additionally, details of medications mentioned in videos were also recorded as “yes” or “no.”

### 2.6. Statistical analysis

Descriptive statistics and nonparametric tests were used to analyze video characteristics and quality scores. The Shapiro–Wilk test was applied to assess the normality of the data. Median and interquartile ranges were used to describe continuous variables. Categorical variables were presented as frequencies and percentages. Chi-square tests or Fisher exact tests were used to compare platform distributions. The Mann–Whitney *U* test was employed to compare interaction data and quality scores between the 2 platforms, while the Kruskal–Wallis test was used for multiple-group comparisons. Where applicable, post hoc comparisons of column proportions were performed, Dunn test with Bonferroni correction was applied to control for multiple testing. Cohen kappa coefficient was used for inter-rater reliability for quality and reliability assessments, with values > 0.8 indicating excellent agreement, 0.6 to 0.8 substantial agreement, 0.4 to 0.6 moderate agreement and < 0.4 poor agreement.^[31]^ Spearman rank correlation coefficient assessed relationships between video general information and quality scores. All statistical analyses were performed using R (version 4.5.0, R Foundation for Statistical Computing, Vienna, Austria). A two-sided *P*-value < .05 was considered statistically significant. All variables were complete for the included videos; therefore, no missing data handling or imputation was required.

## 3. Results

### 3.1. Uploader characteristics

**Figure 2.**
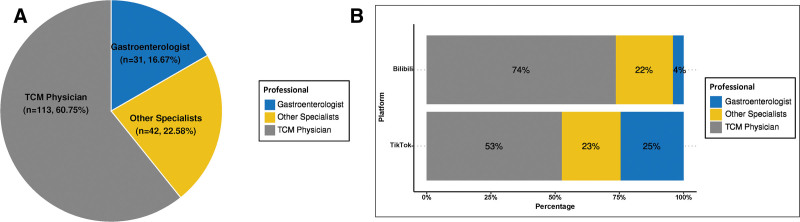
Distribution of professional uploaders. (A) General distribution of professionals on all videos. (B) Distribution of professional uploaders on TikTok and Bilibili. TCM = traditional Chinese medicine.

A total of 214 videos were included in this study, including 124 from TikTok and 90 from Bilibili. Uploader characteristics on the 2 platforms are shown in Table 1. Overall, 86.92% of videos were uploaded by professionals, 11.21% uploaded by nonprofessional individuals and only 4 videos were created by institutions. Professionals remained the main providers of RE-related content on both platforms. There were more videos uploaded by professionals on TikTok (113) than on Bilibili (73). Figure 2 shows the detailed distribution of professional uploaders. A total of 186 videos were created by healthcare professionals, consisting of TCM physicians (60.75%), other specialists (22.67%), and gastroenterologists (16.67%). On TikTok, TCM physicians accounted for 53% of professional uploaders, whereas on Bilibili, other specialists accounted for the largest proportion (52%). Notably, gastroenterologists were not the major contributors on either platform, accounting for only 25% on TikTok and 8% on Bilibili.

**Table 1:** General information, quality scores of RE videos on TikTok and Bilibili.

Variables	Total (n = 214)	TikTok (n = 124)	Bilibili (n = 90)	*P*
Video uploaders				
Professional (n, %)	186 (86.92%)	113 (91.13%)	73 (81.11%)	–
Nonprofessional (n, %)	24 (11.21%)	10 (8.06%)	14 (14.56%)	–
Institution (n, %)	4 (1.87%)	1 (0.81%)	3 (3.33%)	–
General information				
Video length(s), M (Q1, Q3)	98.50 (62.00, 176.65)	76.00 (52.00, 105.00)	167 (103.25, 325.25)	<.001
Likes, M (Q1, Q3)	739.50 (54.00, 3349.25)	2372.50 (878.25, 7015.75)	43.00 (12.25, 147.00)	.002
Collections, M (Q1, Q3)	396.50 (43.50, 2182.00)	1264.50 (400.75, 4438.75)	41.00 (7.25, 134.00)	<.001
Comments, M (Q1, Q3)	70.50 (9.00, 415.50)	310.00 (60.75, 686.75)	8.50 (1.00, 57.25)	<.001
Shares, M (Q1, Q3)	177.50 (14.00, 1417.50)	757.50 (178.25, 3202.00)	14.00 (2.00, 60.75)	.004
Video quality and reliability				
GQS score, M (Q1, Q3)	3 (2, 3)	3 (3, 4)	2 (2, 3)	<.001
mDISCERN score, M (Q1, Q3)	2 (2, 3)	2 (2, 3)	2 (1, 3)	<.001
JAMA score, M (Q1, Q3)	1 (1, 2)	1 (1, 2)	1 (0, 1)	<.001

M: Median, Q1: 1st Quartile, Q3: 3st Quartile.

GQS = Global Quality Score, JAMA = Journal of American Medical, mDISCERN = modified DISCERN, RE = reflux esophagitis.

*P*-value calculated using with Mann–Whitney test, *P* < .05 considered statistically significant.

### 3.2. Video characteristics

Comparative analyses of video length and user engagement metrics (likes, collections, comments, and shares) between the 2 platforms are presented in Table 1. The videos on Bilibili were significantly longer than those on TikTok (167 second vs 76 second, *P* < .001). In contrast, TikTok videos showed substantially higher engagement, including likes (2372.50 vs 43.00, *P* = .002), saves (1264.50 vs 41.00, *P* < .001), comments (310.00 vs 8.50, *P* < .001), and shares (757.50 vs 14.00, *P* = .004). Differences in video length and engagement metrics among uploaders are shown in Table 2. Nonprofessionals uploaded the longest videos length (median 210.5 second) with the lowest engagement. Significant differences in video length and engagement metrics were found across the 3 professional groups (*P* < .001). Videos uploaded by gastroenterologists had the highest engagement, with median likes of 6055, saves of 2190, and comments of 457. Videos by TCM physicians had lower engagement, whereas videos by other specialists showed intermediate levels. Gastroenterologists’ videos had the most shares (median of 2261.5), followed by other specialists (382), and TCM physicians (51).

**Table 2:** Video engagement features of RE videos across different uploaders.

Uploader	Variables				
	Video length(s), M (Q1, Q3)	Likes, M (Q1, Q3)	Collections, M (Q1, Q3)	Comments, M (Q1, Q3)	Shares, M (Q1, Q3)
Nonprofessionals (n = 24)	210.50 (101.75, 392.75)	268.50 (38.50, 1570.25)	130.00 (32.00, 743.25)	70.00 (24.50, 1101.50)	92.00 (13.75, 481.75)
Professionals (n = 186)					
Gastroenterologists (n = 31)	90.00 (61.50, 151.00)	6055.00 (836.00, 9665.00)	2190.00 (462.50, 6510.00)	457.00 (98.50, 1044.50)	2111.00 (221.00, 5458.00)
TCM doctors (n = 113)	99.00 (62.00, 153.00)	222.00 (31.00, 2385.00)	131.00 (20.00, 1421.00)	29.00 (3.00, 191.00)	51.00 (11.00, 633.00)
Other specialists (n = 42)	81.00 (51.00, 190.75)	1477.00 (226.75, 2434.75)	519.50 (181.50, 2236.50)	163.00 (33.25, 364.50)	382.00 (66.75, 1207.75)
*P* *	.0016	.0256	.0867	<.0001	.0145
Effect size r	0.261	0.198	0.161	0.321	0.21
*P*ΔG vs T	.8356	.0187	.0965	<.0001	.0132
*P*ΔG vs O	.0142	.7123	.9234	.0123	.2876
*P*ΔT vs O	<.0001	.1965	.6132	.3654	.7567

M: Median, Q1: 1st Quartile, Q3: 3st Quartile. Effect size r: *R* < 0.10 (minor effect); 0.10 ≤ *R* < 0.30 (small effect); 0.3 ≤ *R* < 0.50 (medium effect); *R* ≥ 0.50 (large effect).

RE = reflux esophagitis, TCM = traditional Chinese medicine.

* *P*-value of Kruskal–Waills test; Δ*P*-value of Dunn test with Bonferroni correction; *P *< .05 considered statistically significant.

### 3.3. Video content

**Figure 3.**
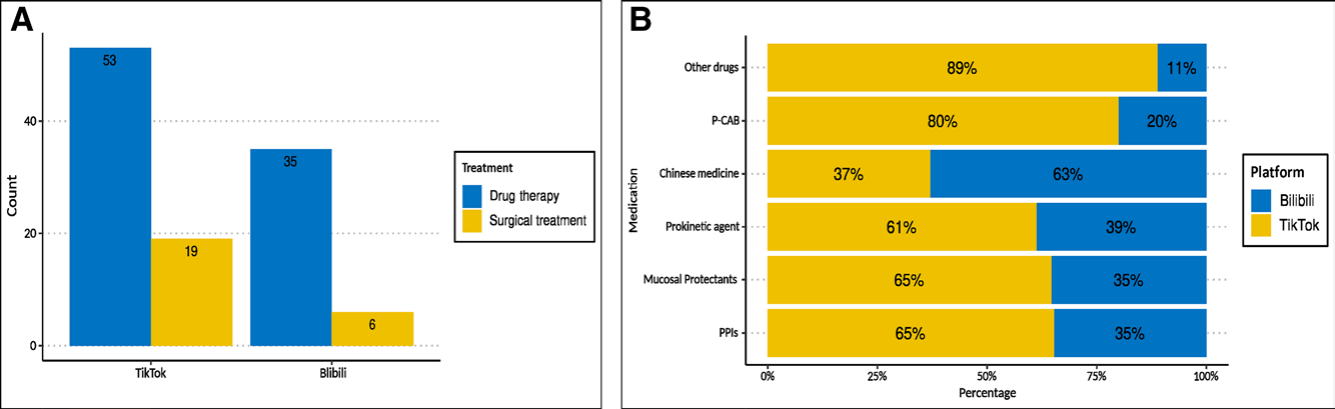
Therapy related content distribution on TikTok and Bilibili. (A) Frequency of mentioned therapy contents on TikTok and Bilibili. (B) Medications mentioned on TikTok and Bilibili. PPIs = proton pump inhibitors, P-CAB = potassium ion competitive acid blocker.

The completeness of video content on 2 platforms is shown in Table 3. Clinical manifestations were the most frequently mentioned topic (75.70%), with 87 videos from TikTok and 75 videos from Bilibili. Epidemiology was the least frequently covered topic (13.55%). The proportions of videos providing detailed clinical manifestations were 47.58% on TikTok and 48.89% on Bilibili. Etiology, treatment, and prognosis were addressed in 124, 113, and 133 videos respectively, with TikTok showing a higher uploading rate than Bilibili. Diagnosis-related content was less common on TikTok (36.29%) than on Bilibili (56.57%). Although the rate of diagnosis content in videos on Bilibili was higher than that on TikTok, most of these videos provide only partial explanations (53.33%) with only 3 full mentioned videos. Overall, the completeness of video content on TikTok was higher than that on Bilibili. Figure 3A shows the distribution of medications mentioned. Moreover, drug therapy was mentioned in 88 videos (53 on TikTok and 35 on Bilibili), and surgical treatment was mentioned in 25 videos. In Figure 3B, detailed medications mentioned in videos included: proton pump inhibitors, mucosal protectants, prokinetic agent, Chinese medicine, potassium ion competitive acid blocker and other drugs. Proton pump inhibitors were frequently featured on TikTok, whereas Chinese medicine was predominantly discussed on Bilibili.

**Table 3:** Completeness content of video content on TikTok and Bilibili.

Contents	Not involved (0 point)		Partial mentioned (1 point)		Full mentioned (2 points)	
	TikTok (n = 124)	Bilibili (n = 90)	TikTok k (n = 124)	Bilibili (n = 90)	TikTok k (n = 124)	Bilibili (n = 90)
Epidemiology, n (%)	107 (86.29)	78 (86.67)	15 (12.1)	12 (13.33)	2 (1.61)	0 (0)
Etiology, n (%)	50 (40.32)	40 (44.44)	36 (29.03)	32 (35.56)	28 (30.65)	18 (20)
Clinical manifestation, n (%)	37 (29.84)	15 (16.67)	28 (22.58)	31 (34.44)	59 (47.58)	44 (48.89)
Diagnosis, n (%)	70 (63.71)	39 (43.33)	40 (32.26)	48 (53.33)	5 (4.03)	3 (3.33)
Treatment, n (%)	53 (42.74)	48 (52.22)	43 (34.68)	29 (33.33)	28 (22.58)	13 (14.44)
Prognosis, n (%)	42 (33.87)	39 (43.33)	45 (36.29)	31 (34.44)	37 (29.84)	20 (22.22)
Sum of score	0	0	207	183	318	196

### 3.4. Video quality

**Figure 4.**
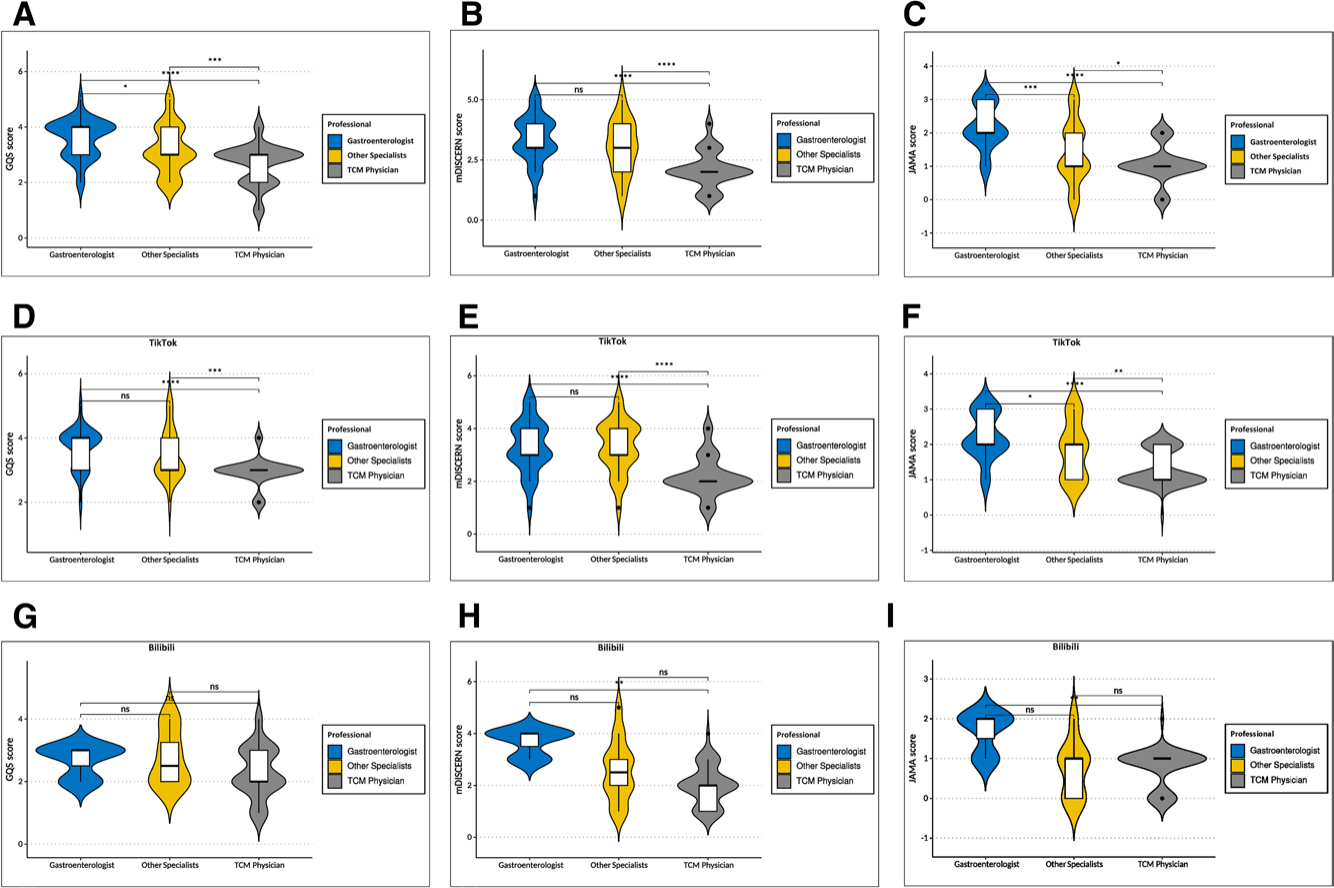
Comparison of quality scores across professional uploaders. (A) Comparison of GQS score among professionals. (B) Comparison of mDISCERN score among professionals. (C) Comparison of JAMA score among professionals. (D) Comparison of GQS score among professionals on TikTok. (E) Comparison of mDISCERN score among professionals on TikTok. (F) Comparison JAMA score among professionals on TikTok. (G) Comparison of GQS score among professionals on Bilibili. (H) Comparison of mDISCER score among professionals on Bilibili. (I) Comparison of JAMA score among professionals on Bilibili. *P < .05, ** *P* < .01, *** *P* < .001, **** *P* < .0001. GQS = Global Quality Score, JAMA = Journal of the American Medical Association, mDISCERN = modified DISCERN.

Video quality and reliability scores for all 214 videos on the 2 platforms are summarized in Table 1. The detailed distribution of quality scores was presented in Table 4. Several recurring patterns of inaccurate or low-quality information were identified (Table S5, Supplemental Digital Content, https://links.lww.com/MD/R319). The Cohen kappa test results showed a high agreement in scoring process with Kappa coefficients of 0.791 for GQS scores, 0.810 for mDISCERN scores, and 0.837 for JAMA scores. The median GQS score was 3 (IQR 2–3), the median mDISCERN score was 2 (IQR 2–3), and the median JAMA score was 1 (IQR 1–2). The median GQS score was 3 (IQR 3–4) for TikTok (59.58% moderate quality and 23.39% good quality) and 2 (IQR 2–3) for Bilibili (44.44% generally poor quality and 33.33% moderate quality). The median mDISCERN score was 2 on both platforms (38.71% on TikTok and 40.00% on Bilibili). JAMA scores were consistently low across both platforms (1 point for 105 videos in total). Significant differences in GQS, mDISCERN, and JAMA scores were observed between TikTok and Bilibili (*P* < .05). As shown in Table 5, nonprofessional creators’ videos got the lowest GQS and mDISCERN score, while most JAMA scores were 0 point. For professional uploaders, gastroenterologists achieved higher scores than other professional groups. The median GQS was 4, mDISCERN score was 3, and JAMA score was 2. In comparison, TCM doctors and other specialists’ videos had lower scores. Comparison of quality scores across 3 healthcare professional groups are presented in Figure 4. Significant differences (*P* < .05) were observed among the professional groups for most quality scores, except for mDISERN score between (Fig. 4A–C). Figure 4D to I showed the detailed quality differences among professionals on 2 platforms respectively. The quality disparity of videos produced by different professionals on TikTok was more apparent, while on Bilibili the difference was less pronounced.

**Table 4:** Detail video quality score distribution on TikTok and Bilibili.

Score	Total (n = 214)	TikTok (n = 124)	Bilibili (n = 90)
GQS score, n (%)			
1	10 (4.67%)	1 (0.81%)	9 (10.00%)
2	56 (26.17%)	16 (12.90%)	40 (44.44%)
3	104 (48.60%)	74 (59.58%)	30 (33.33%)
4	39 (18.22%)	29 (23.39%)	10 (11.11%)
5	5 (2.34%)	4 (3.23%)	1 (1.11%)
mDISERN score, n (%)			
0	4 (1.87%)	3 (2.42%)	1 (1.11%)
1	39 (18.22%)	14 (11.29%)	25 (27.78%)
2	84 (39.25%)	48 (38.71%)	36 (40.00%)
3	50 (23.36%)	31 (25.00%)	19 (21.11%)
4	30 (14.02%)	23 (18.55%)	7 (7.78%)
5	7 (3.27%)	5 (4.03%)	2 (2.22%)
JAMA score, n (%)			
0	43 (20.09%)	10 (8.06%)	33 (36.67%)
1	105 (49.07%)	54 (43.55%)	51 (56.67%)
2	49 (22.90%)	44 (35.48%)	5 (5.56%)
3	17 (7.94%)	16 (12.90%)	1 (1.11%)

GQS = Global Quality Score, JAMA = Journal of American Medical Association, mDISCERN = modified DISCERN.

**Table 5:** Quality scores of RE videos across different uploaders.

Uploader	Variables		
	GQS score, M (Q1, Q3)	mDISCERN score, M (Q1, Q3)	JAMA score, M (Q1, Q3)
Nonprofessionals (n = 24)	2.00 (2.00, 3.00)	1.00 (1.00, 2.00)	0.00 (2.00, 3.00)
Professionals (n = 186)			
Gastroenterologists (n = 31)	4.00 (3.00, 4.00)	3.00 (3.00, 4.00)	2.00 (2.00, 3.00)
TCM doctors (n = 113)	3.00 (2.00, 3.00)	2.00 (2.00, 2.00)	1.00 (1.00, 1.00)
Other specialists (n = 42)	3.00 (3.00, 4.00)	3.00 (2.00, 4.00)	1.00 (1.00, 2.00)
*P* *	<.001	<.001	<.001
Effect size r	0.452	0.541	0.548
*P*ΔG vs T	<.0001	<.0001	<.0001
*P*ΔG vs O	.1123	.4032	.0015
*P*ΔT vs O	<.0001	<.0001	.0876

M: Median, Q1: 1st Quartile, Q3: 3st Quartile. Effect size r: *R* < 0.10 (minor effect); 0.10 ≤ *R* < 0.30 (small effect); 0.3 ≤ *R* < 0.50 (medium effect); *R* ≥ 0.50 (large effect).

RE = reflux esophagitis, TCM = traditional Chinese medicine.

* *P*-value of Kruskal–Waills test; Δ*P*-value of Dunn test with Bonferroni correction.

### 3.5. Spearman correlation analysis

**Figure 5.**
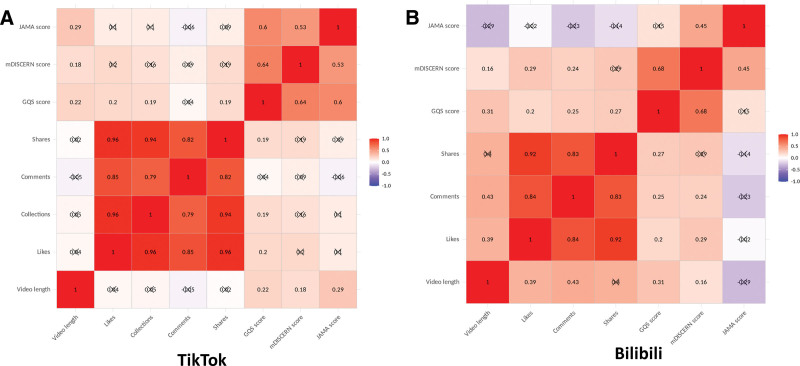
Spearman correlation analysis results between video metrics and quality scores. (A) Spearman correlation analysis results on TikTok (n = 124). (B) Spearman correlation analysis results on Bilibili (n = 90)

As shown in Figure 5, strong positive correlations were observed among engagement metrics (likes, collections, comments, and shares) across both platforms (Spearman *R* ≥ 0.7, *P* < .001). On TikTok, video length showed weak correlation with all 3 quality scores (GQS, mDISCERN, and JAMA), but not with any user engagement metrics. On Bilibili, video length showed moderate correlations with collections (0.3 ≤ Spearman *R* < 0.5, *P* < .001), without correlation with shares or quality scores. Positive correlation between GQS and mDISCERN were found on 2 platforms (*P* < .001). No correlations were observed between engagement metrics and the GQS, mDISCERN, or JAMA scores. The key correlation results (defined as *R* ≥ 0.3 and *P* < .05) by platform with 95% confidence intervals are presented in Table S6, Supplemental Digital Content, https://links.lww.com/MD/R319.

## 4. Discussion

This cross-sectional analysis provides a systematic evaluation of the quality, content structure, and engagement patterns of RE related videos on 2 major Chinese video platforms, TikTok and Bilibili. Rather than merely comparing platforms, our findings offer insights into how professional background, platform ecology, and content framing collectively shapes the quality of dissemination digital health information.

### 4.1. Professional background as a determinant of informational quality

Across both platforms, the professional background of uploaders emerged as a key determinant of video quality and reliability. Videos produced by gastroenterologists consistently outperformed those uploaded by other professional groups and nonprofessionals across GQS, mDISCERN, and JAMA scores in this study. This pattern aligns with previous of evaluations of social media content on gastrointestinal and other chronic diseases,^[22,32,33]^ and underscores the importance of specialty-specific expertise in ensuring clinical accuracy and evidence-based communication.

Importantly, although TCM physicians constituted the largest group of professional content creators, their videos demonstrated lower overall quality scores (GQS, mDISCERN, and JAMA assessment).^[23]^ This discrepancy may not reflect individual competence, but rather differences in conceptual and epistemological frameworks, as well as communication norms, between TCM and modern biomedical systems.^[34]^ Such divergence may complicate standardized evaluation when using assessment tools grounded in evidence-based Western medicine.^[35]^ These findings highlight the need for interdisciplinary dialogue and standardized health communication guidance when diverse medical paradigms coexist on digital platforms.

### 4.2. Platform ecology, engagement, and informational depth

While TikTok’s short-form videos achieved substantially higher user engagement, this increased visibility did not translate into higher informational quality or reliability.^[36]^ Engagement metrics were not correlated with any quality indicators, underscoring the limited usefulness of popularity-based signals for assessing medical accuracy.^[37]^ What is even more thought-provoking is that engagement metrics may further exacerbate the complexity of misinformation propagation, underscoring the necessity for algorithmic adjustments.^[38]^ This reflects a broader structural challenge in social media, where algorithmic prioritization of attention-grabbing content may amplify emotionally appealing or oversimplified narratives over comprehensive health education.^[39]^

Conversely, although Bilibili videos generally longer and provided more detail, greater duration alone did not ensure higher quality scores. This indicates that informational depth depends not merely on video length, but on content organization, source transparency, and adherence to clinical guidelines.^[40,41]^ Collectively, these findings emphasize that neither engagement metrics nor technical production features should be considered as reliable indicators of informational value.^[26,42]^

### 4.3. Content imbalance and implications for patient education

Our content analysis revealed a marked imbalance across major informational domains. Clinical manifestations were emphasized, whereas epidemiology, diagnostic pathways, and prognosis were frequently underrepresented. Such patterns may reflect the relative simplicity of symptom-oriented explanations compared with the complexity of diagnostic criteria or disease progression, particularly in short-format videos. For example, the diagnosis of RE involves endoscopic examination and grading, which can be difficult to convey accurately within limited video time.^[43]^ However, insufficient coverage of diagnostic standards may impair users’ ability to accurately interpret symptoms, seek timely medical evaluation, and adhere to evidence-based management strategies. Additionally, our analysis of treatment content further illustrated platform-specific tendencies. This discrepancies in content may be shaped by to audience preference unique to each platform.^[15,16]^ TikTok videos more frequently addressed pharmacologic therapies consistent with clinical guidelines, whereas Bilibili content more commonly emphasized TCM. These findings may highlight the importance of providing diverse health education content that is divers, culturally sensitive, and scientifically accurate.^[44]^ Although diversity in treatment perspectives may enhance engagement, it remains essential that all therapeutic discussions are presented within accurate risk-benefit frameworks and clearly distinguish supportive care from guideline-recommended interventions.

### 4.4. Reliability gaps and transparency concerns

Despite moderate GQS and mDISCERN scores indicating acceptable informational quality, JAMA scores remained uniformly low across both platforms, highlighting widespread deficiencies in authorship disclosure, source attribution, and content currency.^[45]^ Variability in video quality has also been observed on previous studies, consistent with evaluation results for other healthcare information videos.^[22,24,41]^ These shortcomings have important implications for health literacy, as users may struggle to verify the credibility of information or differentiate evidence-based guidance from opinion-driven content.^[46]^ Enhancing transparency through standardized author identification, evidence citation, and clear update timestamps could substantially improve public trust in digital health communication, particularly in environments where users often rely on heuristic cues rather than formal appraisal of content quality.^[47,48]^

### 4.5. Implications for clinical practice and public health governance

From a clinical perspective, these findings suggest that healthcare professionals should be systematically engaged in digital health education initiatives. Training programs focused on effective science communication may therefore help clinicians balance accuracy with accessibility for diverse audiences.^[49]^ At the platform level, algorithmic optimization strategies that prioritize verified medical content over popularity metrics could reduce the spread of low-quality information.

From a public health standpoint, the development of platform certification or labeling systems for healthcare information videos is warranted.^[40]^ Thus, users are enabled to rapidly identify credible sources. Such measures may be especially valuable in management of chronic diseases such as RE, where sustained adherence to evidence-based management is essential.

## 5. Limitations

Several limitations should be acknowledged. First, this study analyzed only Chinese videos from TikTok and Bilibili, collected at a single time point with limited samples. Consequently, the generalizability of the findings to other languages, regions, platforms, or time periods is limited. Given the dynamic nature of platform recommender systems, which may vary with account history, a single snapshot (August 28, 2025) may not reflect temporal changes in content visibility. Future studies should adopt multi-time point and multi-account sampling to further assess the robustness of the findings.^[50]^ Second, platform specific recommender algorithms prioritize engagement metrics, potentially introducing algorithmic bias by amplifying engaging but less accurate content.^[41]^ Moreover, platform-dependent visibility should also be considered. Cross-platform comparisons are further constrained by differences in moderation policies, community norms and dominant uploader characteristics. In addition, although commonly used evaluation instrument tools were applied, they may not fully capture all aspects of video quality, such as clarity of information delivery and emotional presentation. Finally, users’ knowledge, attitudes or behavioral responses were not assessed. Future studies employing multilingual,^[51]^ multi-platform,^[52]^ multi-tool,^[53]^ and longitudinal approaches,^[54]^ combined with user-based assessments, is warranted.

## 6. Conclusion

This study systematically evaluated 214 RE-related videos on TikTok and Bilibili, integrating analyses of content characteristics, quality and engagement metrics. The results indicate that the overall quality and reliability of available videos were moderate, with significant gaps in content completeness and transparency. Uploader professional background, particularly gastroenterology specialization, was more strongly associated with information quality than popularity metrics. Platform-specific content patterns further suggest that algorithm-driven dissemination does not ensure evidence-based accuracy. These findings underscore the need to promote specialist engagement, enhance transparency standards, and implement evidence-informed platform governance mechanisms. Such measures are essential to improve the quality of health information within digital media environments.

## Acknowledgments

We wish to express our gratitude to no particular individuals for their contributions.

## Author contributions

**Conceptualization:** Zhenzhen Deng, Yueliang Xie, Lu Huang.

**Data curation:** Zhenzhen Deng.

**Formal analysis:** Zhenzhen Deng, Yueliang Xie.

**Investigation:** Yueliang Xie, Shengfeng Wang.

**Methodology:** Zhenzhen Deng, Yueliang Xie.

**Project administration:** Lu Huang.

**Supervision:** Lu Huang.

**Writing – original draft:** Zhenzhen Deng.

**Writing – review & editing:** Yueliang Xie, Shengfeng Wang, Lu Huang.

## Supplementary Material


