# Supplementary material for: Reliability and quality of reflux esophagitis educational videos on TikTok and Bilibili: A cross-sectional study
Source: Medicine (Baltimore). 2026 Feb 6;105(6):e47565. doi: 10.1097/MD.0000000000047565 (PMC12885743; doi:10.1097/MD.0000000000047565)
Supplement: Supplementary file 1 [file medi-105-e47565-s001.docx]

**Supplemental Digital Content 1.** Global Quality Score (GQS) criteria for assessing overall video quality and usefulness.

**Supplementary Table 1** The Global Quality Score (GQS) quality criteria.

| **Item features** | **Points** |
| --- | --- |
| Poor quality; poor flow of the videos; most information missing; not at all useful for patients | 1 |
| Generally poor quality; some information listed, but many important topics missing; of very limited use to patients | 2 |
| Moderate quality; suboptimal flow; some important adequately discussed, but other information poorly discussed; somewhat useful for patients | 3 |
| Good quality and generally good flow; most of the relevant information listed, but some topics not covered; useful for patients | 4 |
| Excellent quality and flow; very useful for patients | 5 |

**Supplemental Digital Content 2.**

Modified DISCERN (mDISCERN) criteria for evaluating the reliability of video-based health information (yes = 1, no = 0).

**Supplementary Table 2** The Modified DISCERN (mDISCERN) quality criteria.

| **Reliability Score*** |
| --- |
| 1. Is the video clear, concise, and understandable? |
| 2. Are valid sources cited? |
| 3. Is the content presented balanced and unbiased? |
| 4. Are additional sources of content listed for patient reference? |
| 5. Are areas of uncertainty mentioned? |

* (1 point for answer ‘yes’, 0 point for answer ‘no’)

**Supplemental Digital Content 3.** Journal of the American Medical Association (JAMA) benchmark criteria for assessing transparency and credibility of video content.

**Supplementary Table 3**. The Journal of the American Medical Association (JAMA) benchmark criteria.

| **Score*** | **Score component** | |
| --- | --- | --- |
| 1 score | Authorship | Author and contributor credentials and their affiliations should be provided. |
| 1 score | Attribution | Clearly lists all copyright information and states references and sources for content. |
| 1 score | Currency | Initial date of posted content and subsequent updates to content should be provided. |
| 1 score | Disclosure | Conflicts of interest, funding, sponsorship, advertising, support, and video ownership  should be fully disclosed. |

*The criteria of each aspect were scored separately, and 1 point for each criterion with a total score of 4 points.

**Supplemental Digital Content 4.** Operational definitions and examples used for standardized evaluation of video content domains.

**Supplementary Table 4** Operational definitions and examples used for video content evaluation

| **Content domain** | **Score category** | **Operational definition** | **Example** |
| --- | --- | --- | --- |
| Epidemiology | Not mentioned (0) | No information regarding affected population, prevalence, or epidemiological characteristics | No mention of who is commonly affected |
|  | Partially mentioned (1) | General or vague description of disease frequency without specifying population or trends | “This disease is very common nowadays.” |
|  | Fully mentioned (2) | Description of affected population, prevalence, or epidemiological trends relevant to the disease | “It mainly affects adults and has become more common in recent years.” |
| Etiology | Not mentioned (0) | No explanation of causes or underlying mechanisms | No mention of disease cause |
|  | Partially mentioned (1) | Single or vague cause mentioned without explanation or mechanism | “It is caused by excess stomach acid.” |
|  | Fully mentioned (2) | Explanation of disease mechanism and major risk factors | “Lower esophageal sphincter dysfunction leads to acid reflux; obesity and alcohol are common risk factors.” |
| Clinical manifestations | Not mentioned (0) | No symptoms described | No symptom mentioned |
|  | Partially mentioned (1) | Only one typical symptom mentioned | “Heartburn is the most common symptom.” |
|  | Fully mentioned (2) | Two or more typical symptoms or inclusion of extra-esophageal manifestations | “Heartburn, acid regurgitation, chest discomfort, or chronic cough.” |
| Diagnosis | Not mentioned (0) | No diagnostic information provided | No mention of diagnosis |
|  | Partially mentioned (1) | Diagnostic method mentioned without explanation of purpose or indication | “Gastroscopy can be used.” |
|  | Fully mentioned (2) | Description of main diagnostic methods and their clinical purpose | “Gastroscopy allows visualization and grading of esophageal mucosal injury.” |
| Treatment | Not mentioned (0) | No treatment information provided | No mention of therapy |
|  | Partially mentioned (1) | Treatment or drug class named without rationale or principle | “Patients should take acid-suppressing drugs.” |
|  | Fully mentioned (2) | Description of major treatment strategies and appropriate use | “Proton pump inhibitors are first-line therapy, combined with lifestyle modification; surgery may be considered in severe cases.” |
| Prognosis | Not mentioned (0) | No discussion of disease outcomes | No mention of prognosis |
|  | Partially mentioned (1) | Vague or generic outcome statements | “The disease is generally controllable.” |
|  | Fully mentioned (2) | Explanation of expected outcomes or potential complications | “Most patients improve with treatment, but uncontrolled disease may increase the risk of complications.” |

**Supplemental Digital Content 5.** Recurring patterns of inaccurate or low-quality information identified in reflux esophagitis-related videos.

**Supplementary Table 5** Recurring Patterns of Inaccurate or Low-Quality Information in RE-Related Videos

| **Pattern** | **Typical Description** | **Potential Implication** |
| --- | --- | --- |
| Oversimplified etiology | RE attributed to a single non-specific cause without pathophysiological explanation | Misunderstanding of disease mechanism |
| Symptom-based self-diagnosis | RE implied based solely on symptoms without clinical evaluation | Overdiagnosis or delayed care |
| Incomplete treatment information | Therapies mentioned without indications, duration, or safety information | Inappropriate self-management |
| Lack of risk disclosure | Absence of adverse effects or contraindications | Increased safety risks |
| Absence of source attribution | No reference to guidelines or evidence sources | Reduced credibility |
|  |  |  |
|  |  |  |

**Supplemental Digital Content 6.**

Key Spearman correlations (with 95% confidence intervals) between engagement metrics and quality scores for TikTok and Bilibili videos.

**Supplementary Table 6** Key Spearman Correlations (with 95% CIs) for TikTok and Bilibili

| **Variable Pair** | **Spearman’s *r*** | **95% CI** | **Correlation Strength** |
| --- | --- | --- | --- |
| **TikTok (n = 124)** |  |  |  |
| Likes ↔ Collection | 0.9605 *** | [0.9441, 0.9722] | Strong |
| Likes ↔ Shares | 0.9584 *** | [0.9411, 0.9707] | Strong |
| Likes ↔ Comments | 0.8506 *** | [0.7933, 0.8930] | Strong |
| Collection ↔ Shares | 0.9355 *** | [0.9091, 0.9544] | Strong |
| Collection ↔ Comments | 0.7862 *** | [0.7081, 0.8454] | Strong |
| Comments ↔ Shares | 0.8207 *** | [0.7535, 0.8710] | Strong |
| GQS ↔ mDISCERN | 0.6376 *** | [0.5197, 0.7316] | Moderate-to-Strong |
| GQS ↔ JAMA | 0.6012 *** | [0.4752, 0.7030] | Moderate-to-Strong |
| mDISCERN ↔ JAMA | 0.5256 *** | [0.3850, 0.6424] | Moderate-to-Strong |
| **Bilibili (n = 90)** |  |  |  |
| Likes ↔ Collection | 0.9635 *** | [0.9448, 0.9759] | Strong |
| Likes ↔ Shares | 0.9237 *** | [0.8858, 0.9493] | Strong |
| Likes ↔ Comments | 0.8473 *** | [0.7760, 0.8972] | Strong |
| Collection ↔ Shares | 0.9514 *** | [0.9267, 0.9679] | Strong |
| Collection ↔ Comments | 0.8125 *** | [0.7273, 0.8731] | Strong |
| Comments ↔ Shares | 0.8355 *** | [0.7594, 0.8891] | Strong |
| GQS ↔ mDISCERN | 0.6796 *** | [0.5491, 0.7778] | Moderate-to-Strong |
| Video length ↔ Comments | 0.4347 *** | [0.2490, 0.5896] | Moderate |
| Video length ↔ Collection | 0.4149 *** | [0.2262, 0.5736] | Moderate |
| Video length ↔ JAMA | -0.3158 | [-0.4917, -0.1151] | Moderate (Negative) |

*** *p* <0.001; Strong: Spearman’s *r*≥0.7, moderate : (0.3≤ Spearman’s *r*<0.5)
